# Comparison between cefepime and carbapenem therapy for deep-seated AmpC-producing *Enterobacterales* infections: a propensity-weighted retrospective cohort study

**DOI:** 10.1128/aac.00928-25

**Published:** 2025-10-31

**Authors:** Nicole Slain, Kristen Lucas, Ashlan J. Kunz Coyne

**Affiliations:** 1 University of Kentucky HealthCare 177468, Lexington, Kentucky, USA; 2 University of Kentucky College of Pharmacy 15511, Lexington, Kentucky, USA; University of Fribourg, Fribourg, Switzerland

**Keywords:** AmpC-producing *Enterobacterales*, inoculum effect, cefepime, meropenem, ertapenem

## Abstract

Deep-seated infections caused by AmpC-producing *Enterobacterales* (AmpC-E) pose challenges due to high bacterial burdens, altered pharmacokinetics, and the inoculum effect. While cefepime and carbapenems are used for bloodstream infections, their comparative effectiveness for deep-seated AmpC-E infections remains uncertain. This retrospective cohort study (2010–2023) evaluated adult inpatients with deep-seated infections caused by AmpC-E (*Enterobacter cloacae* complex, *Klebsiella aerogenes*, or *Citrobacter freundii*). Patients received cefepime or a carbapenem within 48 hours of index culture for ≥72 hours. Definitive therapy (high-dose cefepime or a carbapenem) was administered for ≥70% of the treatment course. The primary outcome was clinical failure, defined as (i) all-cause mortality within 30 days of index culture or (ii) infection recurrence within 30 days after completion of definitive therapy. Pooled logistic regression with inverse probability treatment weighting (IPTW) and time-varying covariates (year of therapy start, time to source control) estimated predictors of failure. Of 480 patients (cefepime *n* = 243, carbapenem *n* = 237), *E. cloacae* complex accounted for 63.9% of infections. Unadjusted 30-day clinical failure rates were similar (14.8% for cefepime vs 11.4% for carbapenem; *P* = 0.267). After IPTW, carbapenem therapy had lower odds of clinical failure (adjusted odds ratio [aOR], 0.44; 95% confidence interval [CI], 0.29–0.83; *P* = 0.033). Intensive care unit (ICU) admission increased failure odds (aOR, 2.39; 95% CI, 1.70–3.35; *P* < 0.001); source control reduced them (aOR, 0.52; 95% CI, 0.40–0.68; *P* = 0.029). Carbapenem therapy is associated with lower adjusted odds of clinical failure than cefepime in deep-seated AmpC-E infections. ICU admission was independently associated with increased odds of clinical failure, while source control was associated with reduced odds of failure.

## INTRODUCTION

Bloodstream infections caused by AmpC-producing *Enterobacterales* (AmpC-E) are clinically challenging due to treatment-emergent resistance and are associated with mortality rates exceeding 20% (1). Chromosomally encoded AmpC genes are found in certain *Enterobacterales* and may be induced by specific β-lactam antibiotics (2). Among these, *Enterobacter cloacae* complex, *Klebsiella aerogenes*, and *Citrobacter freundii* exhibit the highest rates of inducible AmpC resistance, making them moderate-risk species for clinically significant AmpC production (3, 4).

Historically, carbapenems have been the treatment of choice for AmpC-E infections due to their stability against AmpC enzymes and broad-spectrum activity. Cefepime, which is also stable against AmpC β-lactamases, has demonstrated comparable clinical outcomes to carbapenems in treating bloodstream infections (BSI) caused by AmpC-E and is now considered a recommended alternative (4–10). While cefepime and carbapenems show comparable outcomes in AmpC-E BSI, their effectiveness in deep-seated infections, characterized by high bacterial burdens and complex pharmacokinetics, remains understudied.

Deep-seated infections present unique therapeutic challenges, including high bacterial burdens, delayed source control, and altered pharmacokinetics/pharmacodynamics. The inoculum effect, in which increased bacterial density reduces β-lactam efficacy, may amplify AmpC β-lactamase expression, reducing treatment efficacy. Furthermore, identifying the primary infectious source in BSI is often difficult, leading to delays in essential interventions. Prompt and effective source control has been shown to improve outcomes, including mortality, in patients with serious infections (11, 12). To overcome altered drug dynamics in these settings, optimized dosing strategies are critical to ensure adequate antimicrobial exposure (13).

Despite the clinical importance, there is a lack of studies directly comparing cefepime and carbapenems in the treatment of deep-seated AmpC-E infections. This study aims to evaluate clinical outcomes in patients with deep-seated infections caused by moderate-risk AmpC-E species, with or without associated BSI, treated with either cefepime or a carbapenem.

## MATERIALS AND METHODS

This retrospective cohort study was conducted at the University of Kentucky HealthCare system, a two-hospital academic medical center, from January 2010 through December 2023. Adult patients (≥18 years) were eligible if diagnosed with a deep-seated infection caused by *E. cloacae* complex, *K. aerogenes*, or *C. freundii*, confirmed by either (i) microbiological cultures from normally sterile anatomical sites via surgical procedures, incision and drainage, or image-guided aspiration or (ii) a positive blood culture with clinical documentation of a deep-seated infection. Deep-seated infections involved sites such as bone and joint (e.g., osteomyelitis and septic arthritis), deep abscesses in normally sterile compartments (e.g., intra-abdominal, thoracic, and pelvic), or infective endocarditis confirmed by echocardiographic or clinical criteria.

Patients were identified through ICD-9/ICD-10 diagnostic codes for deep-seated infections, cross-referenced with microbiology records. When source cultures were unavailable, inclusion required a positive blood culture and explicit clinical documentation of a deep-seated infection. Eligible patients received cefepime, meropenem, or ertapenem within 48 hours of culture collection and continued therapy for at least 72 continuous hours to ensure early and sustained exposure. For analytic grouping, definitive therapy was defined as the agent administered for ≥70% of the total treatment duration, capturing the predominant treatment course while accounting for potential empiric-to-definitive therapy changes. Included dosing regimens were cefepime 2 g IV every 8 hours, meropenem 2 g IV every 8 hours, or ertapenem 1 g IV every 24 hours, renal dose adjustments based on estimated glomerular filtration rate per institutional protocols. Exclusion criteria included transfer from an outside facility with a known AmpC-E infection, polymicrobial infections, death within 48 hours of diagnosis, and pregnancy or breastfeeding.

The primary outcome was a composite measure of clinical failure, defined as the occurrence of either (i) all-cause mortality within 30 days of index culture collection or (ii) infection recurrence within 30 days after completion of definitive therapy. Infection recurrence was defined as microbiological growth of the same organism from blood or the deep-seated source or by clinical consensus of relapse when cultures were not feasible. Secondary outcomes included all-cause 60- and 90-day mortality, 30-, 60-, and 90-day readmission rates, and hospital length of stay.

Data were collected with assistance from the University of Kentucky Center for Clinical and Translational Science via retrospective chart review and electronic health record extraction. Key definitions were standardized for consistency. The Charlson Comorbidity Index (CCI) was calculated at admission to characterize comorbidities and estimate 1-year mortality risk. Severity of illness was assessed using Acute Physiology and Chronic Health Evaluation II (APACHE II) and Sequential Organ Failure Assessment (SOFA) scores, both calculated within 24 hours of culture collection. Intensive care unit (ICU) admissions were defined by culture collection within 24 hours of ICU entry. Hospital-acquired infections were positive blood cultures collected more than 48 hours post-admission.

Source control was defined as interventions (surgical/percutaneous drainage, debridement, valve replacement/removal) to eliminate the infection focus. Success could be confirmed at any point within 14 days post-intervention, with resolution documented as early as a few days or as late as 14 days. If success was not clearly established within that window, it was reassessed at the end of therapy. Success was determined based on clinical improvement (e.g., fever resolution and reduced inflammatory markers [CRP, ESR, PCT, WBC]), microbiological eradication, radiological evidence of resolution, or clinician consensus. Source control was not required for study inclusion; it was assessed as a clinical variable.

Because no effect-size estimates exist for deep-seated AmpC-E infections, we used prior AmpC-E bacteremia data as a feasibility benchmark, which suggested a minimum cohort of 224 patients to provide 85% power at the 95% confidence level (14). For our study, we prespecified a clinically meaningful absolute difference of 10% in 30-day clinical failure; with cefepime (*n* = 243) and carbapenem (*n* = 237), the final sample size provides 80% power (α = 0.05) to detect ~9%–10% differences, whereas smaller effects may be underpowered. Statistical analyses used χ or Fisher’s exact tests for categorical variables and Student’s *t*-test for continuous/ordinal variables. To address selection bias, propensity scores with inverse probability of treatment weighting (IPTW) were applied. Baseline covariates included age (15), body mass index (16), CCI (17), immunocompromised status (18), SOFA score (19), infectious diseases consult (20), and source control (18).

Time-varying covariates included year of definitive therapy start and time to source control. Final regression variables (ICU admission, hospital-acquired infection, source control, cefepime MIC 4–8 µg/mL [Clinical & Laboratory Standards Institute {CLSI} susceptible dose-dependent] (21), prolonged infusion, and carbapenem therapy) were selected based on univariate *P* ≤ 0.2 or clinical relevance. Covariate balance was assessed using standardized mean differences (<0.1) and collinearity via variance inflation factors (<2). Analyses were conducted using SPSS (version 28.0, Armonk, NY) and R (version 4.3.0, Vienna, Austria). The study received approval from the University of Kentucky institutional review board.

## RESULTS

A total of 480 patients met the inclusion criteria, with 243 (50.6%) receiving definitive therapy with cefepime and 237 (49.4%) receiving a carbapenem (meropenem, 145/237; ertapenem, 92/237). Baseline characteristics were generally similar between groups (Table 1). Notable differences included a higher proportion of ICU admissions in the carbapenem group (cefepime, 124/243 [51.0%] vs carbapenem, 143/237 [60.3%]) and a greater incidence of hospital-acquired infections (cefepime, 91/243 [37.4%] vs carbapenem, 118/237 [49.8%]). The predominant organism was *E. cloacae* complex, accounting for 307/480 (63.9%) of infections (Table 2).

**TABLE 1:** Baseline characteristics^*a*^

Characteristic	Cefepime *n* = 243	Carbapenem *n* = 237	*P* value
Male, *n* (%)	131 (53.9)	126 (53.2)	0.927
Age, years, mean ± SD	54.9 ± 14.2	54.3 ± 14.6	0.601
Race, *n* (%)			0.594
Caucasian	217 (89.3)	216 (91.1)	
African American	23 (9.5)	19 (8.0)	
Other	3 (1.2)	2 (0.8)	
BMI, kg/m^2^, mean ± SD	29.3 ± 9.6	30.4 ± 10.4	0.239
Admit from, *n* (%)			0.210
Non-healthcare origin	159 (65.4)	159 (67.1)	
Other healthcare facility	35 (14.4)	35 (14.8)	
Hospital transfer	35 (14.4)	25 (10.5)	
Clinic office	14 (5.8)	18 (7.6)	
CCI, median [IQR]	4 (2, 6)	4 (3, 7)	0.898
APACHE II score, median [IQR]	19 (15, 22)	18 (15, 22)	0.367
SOFA score, median [IQR]	5 (3, 10)	5 (4, 10)	0.944
ICU admission, *n* (%)	124 (51.0)	143 (60.3)	0.040
Immunocompromised,^*b*^ *n* (%)	70 (28.8)	73 (30.8)	0.633

^*a*^ SD, standard deviation; BMI, body mass index; CCI, Charlson comorbidity index; IQR, interquartile range; APACHE II, Acute Physiology and Chronic Health Evaluation II score; SOFA, sequential organ failure assessment; ICU, intensive care unit.

^*b*^ Immunocompromised: any chemotherapy or radiation therapy within 30 days, HIV/AIDS with a CD4 <200, or chronic steroids (equivalent to >40 mg prednisone).

**TABLE 2:** Infection characteristics^*a*^

Characteristic	Cefepime *n* = 243	Carbapenem *n* = 237	*P* value
Definitive antibiotic, *n* (%)			<0.001
Cefepime	243 (100.0)	0 (0.0)	
Meropenem	0 (0.0)	145 (61.2)	
Ertapenem	0 (0.0)	92 (38.8)	
Prolonged infusion^*b*^, *n* (%)	138 (56.8)	68 (46.9)	0.059
Hospital acquired infection^*c*^, *n* (%)	91 (37.4)	118 (49.8)	0.006
Infectious diseases consult, *n* (%)	200 (82.3)	204 (86.1)	0.264
Organism, *n* (%)			<0.001
*E. cloacae* complex	132 (54.3)	175 (73.8)	
*K. aerogenes*	59 (24.3)	41 (17.3)	
*C. freundii*	52 (21.4)	21 (8.9)	
Cefepime MIC, *n* (%)			
≤0.5	77 (31.7)	57 (24.1)	0.062
1	66 (27.2)	59 (24.9)	0.572
2	75 (30.9)	87 (36.7)	0.176
4	16 (6.6)	14 (5.9)	0.759
8	9 (3.7)	20 (8.4)	0.064
CSDD^*d*^	25 (10.3)	34 (14.3)	0.175
Source of infection, *n* (%)			
Bone and joint	114 (46.9)	130 (54.9)	0.171
Deep abscess	127 (52.3)	104 (43.9)	0.191
Endocarditis	2 (0.8)	3 (1.3)	0.736
Source control success^*e*^, *n* (%)	131 (53.9)	145 (61.2)	0.117
Concomitant blood infection with index organism	69 (28.4)	53 (22.4)	0.129
Duration of total therapy^*f*^, days, median (IQR)	23 [16, 29]	26 [20, 33]	0.668
Bone and joint	29 [21, 37]	33 [25, 41]	
Deep abscess	23 [16, 30]	21 [15, 28]	
Endocarditis	41 [34, 50]	39 [31, 47]	

^*a*^ MIC, minimum inhibitory concentrations; CSDD, cefepime susceptible dose dependent; IQR, interquartile range.

^*b*^ Prolonged infusion analysis excludes ertapenem due to its standard once-daily 30-minute infusion.

^*c*^ Hospital-aquired infection: index positive blood culture collected 48 hours after hospital admission.

^*d*^ Cefepime MIC value of 4–8 µg/mL.

^*e*^ Source control: procedural intervention to eliminate the infectious focus (surgical/percutaneous drainage, debridement, or valve replacement/removal). “Success” was confirmed within 14 days post-intervention, or, if indeterminate, reassessed at end of therapy, based on any of the following: clinical improvement (e.g., defervescence, decreasing CRP/ESR/PCT/WBC), microbiologic eradication, radiologic resolution, or clinician consensus.

^*f*^ Duration of total therapy accounts for inpatient and outpatient therapy of cefepime, meropenem, and ertapenem.

Unadjusted 30-day clinical failure rates were comparable between groups (cefepime, 14.8%, vs carbapenem, 11.4%, *P* = 0.267), as were 60-day mortality (cefepime, 8.2%, vs, carbapenem, 11.4%, *P* = 0.244) and 90-day mortality (cefepime, 9.1% vs, carbapenem, 13.1%, *P* = 0.159). Median hospital length of stay was shorter in the cefepime group (11 days [IQR 6-23]) compared with the carbapenem group (14 days [IQR 7-31]; *P* = 0.041). The 30-day readmission rate was numerically lower in the cefepime group (cefepime, 9.1%, vs carbapenem, 13.2% ; *P* = 0.159), while 60-day (cefepime, 2.5% vs carbapenem, 3.8%, *P* = 0.403) and 90-day readmission rates (cefepime, 2.9%, vs carbapenem, 4.2%, *P* = 0.428) were similar (Table 3).

**TABLE 3:** Clinical outcomes^*a*^

Characteristic	Cefepime *n* = 243	Carbapenem *n* = 237	*P* value
Clinical failure, *n* (%)	36 (14.8)	27 (11.4)	0.267
All-cause 30-day mortality	27 (11.1)	24 (10.1)	0.726
30-day infection recurrence	9 (3.7)	3 (1.3)	0.087
All-cause mortality, *n* (%)			
60-day	20 (8.2)	27 (11.4)	0.244
90-day	22 (9.1)	31 (13.1)	0.159
Re-admission rate, *n* (%)			
30-day	22 (9.1)	31 (13.1)	0.159
60-day	6 (2.5)	9 (3.8)	0.403
90-day	7 (2.9)	10 (4.2)	0.428
Hospital length of stay, days, median [IQR]	11 (6, 23)	14 (7, 31)	0.041

^*a*^ IQR, interquartile range.

In the propensity-weighted logistic regression model, adjusted for relevant clinical and temporal confounders, definitive carbapenem therapy was independently associated with significantly lower odds of clinical failure (adjusted odds ratio [aOR] 0.44, 95% confidence interval [CI] 0.29–0.83; *P* = 0.033). ICU admission (aOR 2.39, 95% CI 1.70–3.35; *P* < 0.001) was linked to increased odds of failure, while successful source control was protective (aOR 0.52, 95% CI 0.40–0.68; *P* = 0.029) (Table 4).

**TABLE 4:** Propensity-weighted logistic regression for clinical failure with time-varying covariates in AmpC-E^*a*^

Variable	OR (95% CI)	*P* value	aOR (95% CI)	*P* value
ICU admission	5.29 (1.93–14.43)	0.001	2.39 (1.70–3.35)	<0.001
Hospital-acquired infection	0.92 (0.40–2.10)	0.841	0.99 (0.91–1.08)	0.148
Prolonged infusion^*b*^	0.79 (0.32–1.58)	0.401	0.74 (0.60–1.02)	0.236
Definitive carbapenem treatment	0.58 (0.31–1.10)	0.094	0.44 (0.29–0.83)	0.033
Source control	0.60 (0.27–1.33)	0.211	0.52 (0.40–0.68)	0.029
Cefepime SDD MIC	0.71 (0.21–2.34)	0.573	1.86 (0.99–2.75)	0.091

^*a*^ OR: odds ratio; confidence interval, CI; aOR: adjusted odds ratio; ICU, intensive care unit; SDD MIC, susceptible dose-dependent minimum inhibitory concentration.

^*b*^ Prolonged infusion applies to cefepime and meropenem only; ertapenem is given as a standard 30-minute once-daily infusion. In regression models, ertapenem was coded 0 (standard infusion).

## DISCUSSION

This study evaluated the comparative effectiveness of high-dose cefepime versus carbapenems for deep-seated infections caused by AmpC-E. Unlike prior investigations that primarily focused on bloodstream infections or included deep-seated infections within broader cohorts, our analysis specifically addressed infections characterized by high bacterial burden and complex pharmacokinetics. After adjustment using IPTW and time-varying covariates, definitive carbapenem therapy was independently associated with lower odds of 30-day clinical failure compared with cefepime (aOR, 0.44; 95% CI, 0.29–0.83; *P* = 0.033), suggesting greater clinical effectiveness in this context. Although crude and secondary analyses were largely null and may reflect residual confounding, IPTW adjustment accounted for measured differences and provided a more valid estimate of the association between carbapenem therapy and reduced failure.

Our findings contrast with several AmpC-E bloodstream infection studies reporting similar outcomes between cefepime and carbapenems (6–10, 14, 22, 23). Deep-seated infections present unique challenges, such as higher inocula and limited tissue penetration, that can blunt cefepime activity (7, 9, 24, 25). In our cohort, carbapenem recipients more often had ICU admissions and hospital-acquired infections, indicating greater baseline severity, yet APACHE II and SOFA scores were similar between groups. Despite these imbalances, carbapenem therapy remained independently associated with lower adjusted odds of clinical failure. By contrast, 30-, 60-, and 90-day mortality were similar between groups, indicating that the observed treatment effect was driven primarily by the composite clinical failure endpoint rather than mortality alone. Our focus on deep-seated infections, and use of high-dose cefepime may partly explain differences from studies that included mixed infection sites or standard-dose cefepime. Although current guidance supports cefepime as a carbapenem-sparing option for AmpC-E (4), our adjusted findings suggest a possible advantage of carbapenems in deep-seated disease. These results should be interpreted cautiously given potential residual confounding, local practice patterns, and microbiology differences, and they do not preclude cefepime use in carefully selected patients with effective source control and susceptible isolates. Prospective multicenter studies are needed to determine when cefepime is comparable and when carbapenems are preferred.

One possible explanation for the adjusted association is greater pharmacodynamic stability of carbapenems at high inoculum, whereas cefepime may be more susceptible to the inoculum effect. *In vitro* studies have demonstrated that cefepime is particularly vulnerable to this phenomenon in AmpC-producing organisms. For example, in a study of 62 clinical *Enterobacter* isolates, a 10-fold increase in inoculum raised the cefepime MIC₉₀ from 8 to 256 mg/L, with more than 60% of isolates exhibiting eightfold or greater MIC increases and nonsusceptibility rates rising from 6.5% to 54.8% (26). Similar findings have been reported in *E. cloacae*, *K. aerogenes*, and *C. freundii* (27). These pharmacologic limitations may contribute to the reduced clinical effectiveness of cefepime in high-burden, deep-seated infections. In such settings, repeat cultures are often infeasible due to the invasive nature of the infection sites, potentially masking rising MICs during treatment. This could help explain the low recurrence rates observed in both treatment groups, although the higher 30-day recurrence in the cefepime group (3.7% vs 1.3%, *P* = 0.087) may reflect undetected microbiologic persistence resulting from the reduced activity of cefepime in some cases. Routine phenotypic susceptibility testing may not reliably identify underlying resistance mechanisms, such as AmpC derepression or ESBL coexpression, which can delay recognition of reduced cefepime activity in deep-seated, high-inoculum infections. In this context, empiric therapy should account for diagnostic uncertainty early in the course, with prompt de-escalation once mechanisms are excluded and source control is achieved. These limitations highlight the value of rapid genotypic testing to guide therapy.

Source control was strongly associated with lower odds of clinical failure (aOR, 0.52; 95% CI, 0.40–0.68; *P* = 0.029). Although achieved more often in the carbapenem group (61.2% vs 53.9%; *P* = 0.117), this difference was not statistically significant, though the absolute difference could still have influenced outcomes. The protective effect aligns with prior evidence on timely source control in complex infections, including intra-abdominal abscesses and osteomyelitis (11, 12). Notably, the adjusted association favoring carbapenems persisted after accounting for source control and calendar time, but given the retrospective, single-center design, these findings should be interpreted cautiously and as hypothesis-generating rather than prescriptive. Defining source control remains challenging. Some procedures, such as valve replacement or amputation, are definitively eradicative, whereas others rely on indirect markers such as clinical improvement or lack of radiographic progression. Sterile follow-up cultures are often not feasible at deep sites, which can introduce variability and potential misclassification. Operationally, source control was assessed within a 14-day window after intervention and was reassessed at the end of therapy if initially indeterminate, which introduces heterogeneity in the timing of adjudication. Moreover, source control was evaluated separately from the 30-day composite outcome of clinical failure, but overlap between these constructs may influence interpretation. Because our categorization combined microbiologic, clinical, and procedural endpoints, and because negative cultures and clinical resolution are not equivalent markers of eradication, misclassification of source control status is possible and could have affected observed associations.

This retrospective, single-center design is subject to several additional limitations. Patients receiving carbapenems more often had ICU admissions and hospital-acquired infections, which may indicate greater underlying illness severity and could have introduced treatment selection bias. Two temporal factors may also have influenced agent selection: revisions to cefepime breakpoint interpretations during the study period and the institution’s adoption of high-dose, extended-infusion cefepime. To address secular trends, the IPTW model included time-varying covariates for year of definitive therapy initiation and time to source control, but prescriber rationale was not systematically captured, and species-level differences may leave residual confounding. The adjusted association favoring carbapenems persisted after these adjustments, but causality cannot be inferred from a single-center retrospective cohort. Length of stay was longer in the carbapenem group (14 vs 11 days, *P* = 0.041), and 30-day readmission was numerically higher (13.2% vs 9.1%, *P* = 0.159), findings that may reflect underlying severity rather than a treatment effect, consistent with prior work linking ICU admission and nosocomial infection to readmission risk (28, 29). Potential microbiome effects of broad-spectrum therapy, including *Clostridioides difficile*, also warrant prospective evaluation.

Reliance on phenotypic susceptibility testing limited detection of specific β-lactamase mechanisms, including possible ESBL coexpression, which could have influenced outcomes. Although blood isolates with confirmed CTX-M genes were excluded, broader genotypic screening was not performed. Prior work has questioned the reliability of cefepime when unrecognized β-lactamases are present, and a US analysis of *E. cloacae*, *K. aerogenes*, and *C. freundii* found ESBL genes in 7% of isolates with cefepime MIC ≤2 µg/mL and in 67% with MIC ≥16 g/mL, with none detected in the 4–8 µg/mL range (30).

These data underscore the limits of phenotype-only approaches and support incorporating genotypic testing when feasible. Cefepime may remain reasonable in carefully selected cases with susceptible isolates and effective source control, but MIC results should be interpreted cautiously and in clinical context. Cefepime MIC values of 4–8 µg/ml (the CLSI susceptible dose-dependent range) were included as covariates in our models but were not analyzed as subgroups, which may have limited our ability to identify differential outcomes in this range. Therapeutic drug monitoring was not performed, and suboptimal cefepime exposure may have contributed to failures (28, 29). Combination therapy for the small Gram-negative infective endocarditis (IE) subgroup (*n* = 5) was not captured, limiting interpretation of outcomes for these patients. Initial therapy within the first 24–72 hours may have differed from definitive therapy, and alternative agents used in this window were not systematically recorded. Because recurrence was assessed only after completion of definitive therapy, patients who died during treatment were not at risk for this outcome, introducing potential immortal time bias. Finally, the study was not prospectively powered for deep-seated infections. Although the sample size was adequate to detect an approximate 10% absolute difference in 30-day clinical failure, smaller effects may have been missed, and Type II error remains possible, particularly for secondary outcomes.

The study was not designed to directly compare meropenem with ertapenem. Although 38.8% of carbapenem recipients received ertapenem for at least 70% of total treatment duration, sequencing earlier in the course was not captured. Some patients likely received cefepime or meropenem initially with ertapenem started later, after much of the acute management had occurred. As a result, the data do not clarify whether meropenem or ertapenem is preferred in the initial phase of deep-seated AmpC-E infection, nor the optimal timing for transition to ertapenem. Dedicated comparative studies are needed. The multiyear study period may also have introduced changes in local practice, guideline interpretations, and resistance patterns that could have influenced agent selection and treatment duration, and these temporal effects warrant prospective evaluation.

In this single-center retrospective cohort of deep-seated AmpC-E infections, carbapenem therapy was associated with lower IPTW-adjusted odds of clinical failure compared with high-dose cefepime. This association may relate to greater pharmacodynamic stability at high inoculum, but causality cannot be inferred. Cefepime may remain appropriate in carefully selected patients with susceptible isolates and effective source control. Management should be individualized based on infection source, organism burden, and resistance considerations. Prospective studies that incorporate genotypic resistance testing and therapeutic drug monitoring are needed to refine agent selection, treatment duration, and sequencing strategies.
